# Carbon storage and sequestration potential in aboveground biomass of bamboos in North East India

**DOI:** 10.1038/s41598-020-80887-w

**Published:** 2021-01-12

**Authors:** Angom Sarjubala Devi, Kshetrimayum Suresh Singh

**Affiliations:** Department of Environmental Science, Mizoram University, Aizawl, 796004 India

**Keywords:** Ecology, Environmental sciences

## Abstract

The Northeastern hilly states of India harbor nearly 90 species of bamboos, 41 of which are endemic to the region. Estimation of C-storage and C-sequestration in aboveground biomass of two common bamboo species namely *Bambusa tulda* and *Dendrocalamus longispathus* was carried out in Mizoram-one of the eight states of Northeastern India. Recording of density of culms was done by quadrate method and harvesting of culms was done to estimate the aboveground biomass. C-storage in different components of the culms was found out for three age classes namely 1, 2 and ≥ 3 year old culms. Aboveground biomass ranged from 73.58 to 127 Mg/ha in *Bambusa tulda* and 115 to 150 Mg/ha in *Dendrocalamus longispathus*. Culm density and aboveground biomass were maximum in the ≥ 3 year age class in both the species. C-storage ranged from 36.34 to 64.00 Mg/ha in *Bambusa tulda* and 50.11 to 65.16 Mg/ha in *Dendrocalamus longispathus*. Although having lower aboveground biomass the rate of C-sequestration was higher in *Bambusa tulda* with 27.79 Mg/ha/year than *Dendrocalamus longispathus* which have 15.36 Mg/ha/year. The reason was attributed to higher increment of culm density and DBH of the older age class in the second year study period in *Bambusa tulda*.

## Introduction

Bamboos occupy 13% of the total forest area of India. A total of 145 species belonging to 23 genera were reported from India^1^. Maximum concentration of bamboo species are found in the deciduous and semi-evergreen regions of Northeast and tropical moist deciduous forests of North and South India. About 70% of bamboo species were reported from Northeast and Western Ghats. The Northeastern hilly states harbor nearly 90 species of bamboos, 41 of which are endemic to this region^2^. Out of the eight sister states of Northeast India Mizoram recorded 35 bamboo species^3^. *Bambusa tulda* Roxb. locally known as *Rawthing* is native in Northeastern states of India and the neighboring countries Myanmar, Thailand and Bangladesh. It can attain upto 15–20 m tall and can grow in moderate to steep slopes. *Dendrocalamus longispathus* (Kurz) Kurz. locally known as *Rawnal* is native to the present study area Mizoram one of the eight states of Northeastern India. It can grow upto 12-20 m tall and prefers mostly steep slopes having 40–70% slope^4^. Both the bamboos have sympodial growth habit. Their young shoots are edible and are available plenty in the market during rainy season. Among the two, young shoots of *Dendrocalamus longispathus* are more preferred by the local people. Due to their thick walled structure they are used for construction of houses, for making baskets, mats, water vessels, furniture, toys and hats. Bamboo is an indispensable resource of the rural population in Northeast India. Due to shifting cultivation the sympodial bamboo forests area is declining and monopodial bamboo especially *Melocanna baccifera* have dominated as it can easily adapt in fallows. Initiatives have been taken up by the state government to use the potential of bamboo forest by establishing bamboo based small scale industries in different districts. Among the policies carbon trading mechanism through plantation of bamboos in degraded lands can be initiated as bamboo has fast growing tendency. Biomass accumulation potential of bamboo is much greater than most fast-growing exotic tree species of highland areas. Its ability to grow fast and existence for a long period of time without causing a significant change in the culm stock after harvesting makes bamboo one of the potential and priority species for carbon storage and sequestration^5^. Many studies have reported rate of carbon sequestration from different types of bamboos^6–10^. However very less work on carbon storage and C-sequestration in aboveground biomass of *Bambusa tulda* and *Dendrocalamus longispathus* have been reported from the state. Therefore, the present work was undertaken to assess the C-storage in aboveground biomass of different age class of culms and capability of C-sequestration in the two types of bamboo species in order to provide a baseline information for future assessment.

## Materials and methods

### Study site

The study was carried out in Mizoram Bamboo Centre located in Lengpui, Mizoram, one of the eight states of Northeast India. The study site has varied terrain feature ranging from gentle to very steep slopes. The center was established in the year 2007 under the initiative of National Bamboo Mission. Plantation of different species of bamboo was undertaken in the center for conservation. At present the center has established as a protected bamboo forests covering an area of approximately 15 hectares.

In the month of March, 2016 three plots having sizes of 0.25 ha each were selected randomly each for *Bambusa tulda* Roxb. (BT) and *Dendrocalamus longispathus* (Kurz) Kurz. (DL) making a total of 6 plots and a total area of 1.5 ha. The study site has altitude of 380–325 m a.s.l. and located at latitude of 23° 83′ 32″ N and longitude of 92° 62′ 63″ E. The area is characterized by a subtropical monsoon climate with three distinct seasons—Summer (March–May), Rainy (June–September) and Winter (November–February), October is the transition month between Rainy and Winter seasons. The average maximum temperature for the years 2016 and 2017 was 28.09 °C and average minimum temperature was 11.25 °C. The average annual rainfall amounts to 288.08 cm with 85% concentrated during Rainy season^11^. The soil was yellowish red in color which is equivalent to Inceptisols according to Soil Survey Staff, USDA^12^. Soil samples were collected randomly from the two types of bamboo stands from a depth of 0–30 cm after removing the litter layer. The soil was homogenized manually and maintained three replicates. Soil pH was determined by 1:2.5 soil water solution using Potentiometer and organic C was analyzed using Walkley and Black’s titration method^13^. Soil pH was recorded to be 5.5 ± 0.01 and organic C to be 2.40 ± 0.33%. Enumeration for culm density and harvesting of culms for biomass estimation was carried out annually after rainy season during October and November during the years 2016 and 2017.

### Aboveground biomass

In each of the three plots selected three quadrates having sizes of 100 m^2^ were laid. Bamboo culms within the quadrates were classified into three different age classes namely 1 year, 2 year and ≥ 3 year old age class. The classification of age class was done based upon Banik^14^. Number of culms in each age class for each quadrate were recorded for finding culm density. Diameter at breast height (DBH) for the culms were recorded at 1.3 m above the ground. Three culms from each age class were harvested from each quadrate. 27 culms were harvested in 1 year for each bamboo species. Each harvested bamboo was classified into sheath, culm, branch and leaf components. Fresh weight of the different components of each of the harvested culms was determined in the field and recorded. Sub-samples of each component were brought to the laboratory and oven dried at 103 °C for 48 h in order to found out dry weight. The total dry weight for different components of each bamboo culm was calculated by following the method given by FAO^15^.

$$TDW=TFW \frac{SDW}{SFW}$$

where TDW = total dry weight, TFW = total fresh weight, SDW = sample dry weight and SFW = sample fresh weight.

The weight of all the different components were summed up in order to give biomass of each culm. Total aboveground biomass of each bamboo species for each age class was determined separately by multiplying the average biomass of the culms in each age class with their respective culm densities.

### Litter-fall collection

Permanent trays having sizes of 1 m^2^ were laid down in the earmarked plots for collection of fallen litter. The litter fallen were collected on seasonal basis for the 2 years. Collected litterfall was separated into different components of sheath, branch and leaf. The components were oven dried separately for finding biomass.

### C-content

Analysis of percentage C content for all the components of harvested culms and litterfall was determined by the help of CHNS-Analyzer.

### C-storage and C-sequestration

The C-storage in each components of aboveground biomass of each age class and litterfall was determined by multiplying biomass with their respective percent C-content divided by 100. Total C-storage in aboveground biomass for each year was found out by summing up the biomass of different age classes. C-storage in litterfall for each year was also determined.

C-sequestration in aboveground biomass was calculated by: C-sequestration = C-storage_2017_ − C-storage_2016_ + C-storage_litterfall_^16^.

### Statistical analysis

All the data were subjected to analysis of variance, correlation between different components of biomass with DBH and culm density was determined and t test was performed for the correlation results. Statistical analysis was carried out by using SPSS version 25 and Excel Microsoft.

## Results

### DBH and culm density

**Figure 1:**
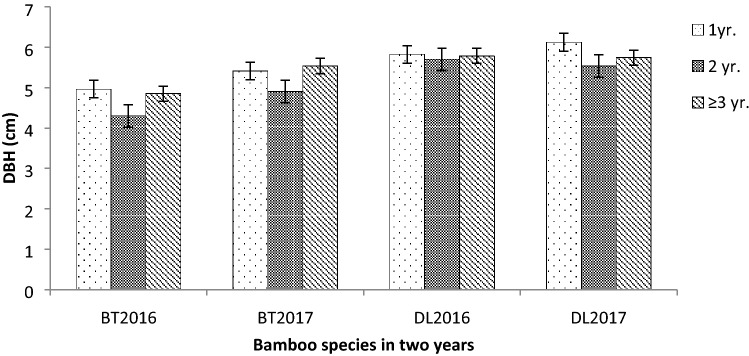
DBH of BT and DL under different age class (± SE).

**Figure 2:**
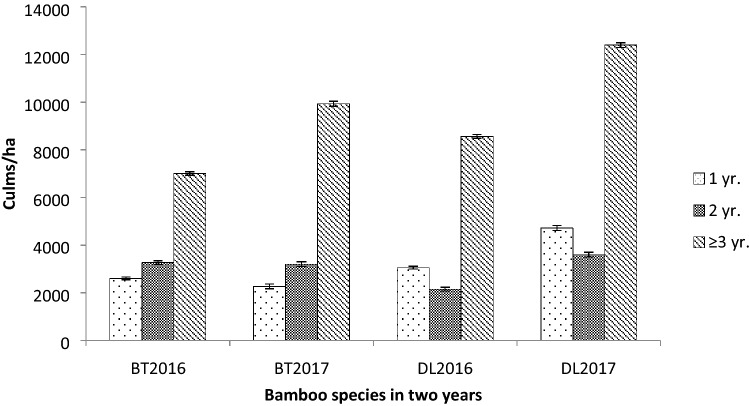
Culm densities of BT and DL under different age class (± SE).

The DBH in BT ranged from 4.30 to 5.53 cm and 5.53 to 6.12 cm in DL (Fig. 1). Average DBH was 4.95 cm and 5.70 cm in BT and DL, respectively. There was no significant variation of DBH in all the age classes in both the species and in both years. However the 2 year old culms showed a decline in their sizes compared with the other two age classes. The culm density of BT showed significant variation in the 2 years (F_2,15_ = 67.01, p < 0.05) as well as in DL (F_2,15_ = 10.42, p < 0.05). The average total culm density for the 2 years of DL was higher with 17,240 culms/ha than BT which had 14,132 culms/ha (Fig. 2). In BT density of 1 year old culms was minimum whereas in DL the density of 2 year old culms was minimum. Maximum culm density was observed in ≥ 3 year old culms in both bamboos.

### Aboveground biomass

**Figure 3:**
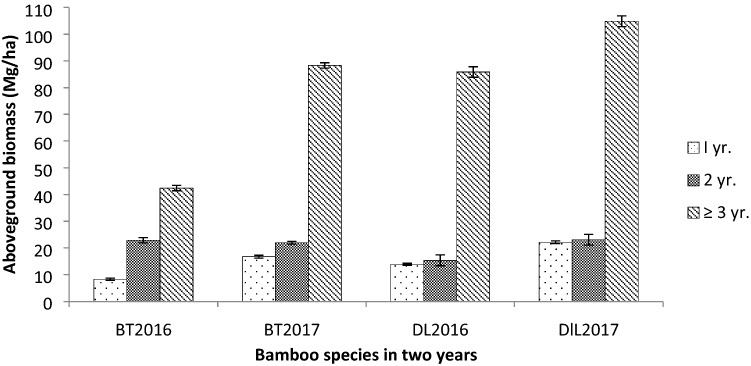
Aboveground biomass of BT and DL in different age class (± SE).

In both the species presence of sheath was recorded in the 1 year old culms only. Absence of leaves and branch was observed in 1 year old culms of BT. The average contribution of different components in total aboveground biomass of BT was 84% by culm, 8.1% by branch and 7.1% by leaves. In DL it was 86% by culm, 8.7% by branch and 5% by leaves. Significant variation of culm biomass (F_2,24_ = 108, p < 0.01) and leaf biomass (F_2,18_ = 47, p < 0.01) was obtained during 2016 in BT. During 2017 significant variation was also observed in culm (F_2,24_ = 467, p < 0.01), branch (F_2,18_ = 64, p < 0.01) and leaf biomass (F_2,18_ = 196, p < 0.01) in BT. In DL significant variation of culm biomass (F_2,24_ = 51, p < 0.01), branch (F_2,24_ = 202, p < 0.01), and leaf biomass (F_2,24_ = 21, p < 0.05) was obtained during 2016. During 2017, significant variation was observed in culm (F_2,24_ = 202, p < 0.01) and branch biomass (F_2,24_ = 21, p < 0.01) during 2017. Maximum contribution to aboveground biomass was recorded by the ≥ 3 year old age class in both the bamboos (Fig. 3). The overall contribution of biomass to the total aboveground biomass by 1 year old age class was 12%, 22% by 2 year old and 65% by ≥ 3 year old in BT. In DL 13% by 1 year old, 14% by 2 year old and 72% by ≥ 3 year old age classes. The total aboveground biomass was 74 Mg/ha during 2016 and 127 Mg/ha during 2017 in BT. In DL it was 115 Mg/ha during 2016 and 150 Mg/ha during 2017.

Significant positive correlation of aboveground biomass with DBH was observed only in BT however with culm density significant positive correlation was observed in both the bamboos in both the years (Table 1).

**Table1:** Pearson’s co-efficient of correlation (r, n = 9) of aboveground biomass with DBH and culm density (**indicates significance at p < 0.01, ^ns^ indicates no significance).

	DBH	Culm density
**Aboveground biomass of BT**		
2016	0.56**	0.96**
2017	0.60**	0.99**
**Aboveground biomass of DL**		
2016	0.17^ns^	0.97**
2017	0.17^ns^	0.98**

### Litterfall

Maximum contribution by leaf component was observed in both the bamboos in litterfall component for both the years (Table 2). Total biomass in litterfall was 0.47 Mg/ha and 0.54 Mg/ha in BT and DL, respectively.

**Table 2:** Litterfall in BT and DL (± SE).

Year	Components	BT (Mg/ha)	DL (Mg/ha)
2016	Leaf	0.07 ± 0.001	0.09 ± 0.001
	Branch	0.06 ± 0.0009	0.07 ± 0.0008
	Sheath	0.07 ± 0.001	0.10 ± 0.002
2017	Leaf	0.11 ± 0.0013	0.12 ± 0.001
	Branch	0.07 ± 0.0004	0.07 ± 0.0003
	Sheath	0.09 ± 0.002	0.09 ± 0.001

### C-content

The C-content was found to be highest in branch (53.12%) followed by culm (49.4%) and leave component (45%) in BT whereas in DL the highest percentage of C was found in culm (42.81%) followed by branch (42.79%) and leave component (41.43%) (Table 3). No significant variation for C-content was observed for both the species in the different age classes. Average C-content in sheath of BT and DL were found to be 30.14% and 29.14%, respectively. Between the two bamboo species, BT was found to have higher amount of C-content with an average of 46.83% and 41.02% in DL.

**Table 3:** C-content (%) in different components of BT and DL (± SE).

Age class	BT				DL			
	Culm	Branch	Leaf	Sheath	Culm	Branch	Leaf	Sheath
1 year	48.50 ± 0.9	–	–	30.14 ± 0.45	44.50 ± 0.06	43.56 ± 0.56	41.60 ± 1.11	29.14 ± 0.45
2 year	47.82 ± 1.11	54.75 ± 3.75	45.75 ± 0.75	–	39.58 ± 1.24	40.72 ± 0.23	42.60 ± 0.55	–
≥ 3 year	52.00 ± 1.25	51.50 ± 1.25	44.25 ± 0.87	–	44.35 ± 0.15	44.11 ± 1.23	40.11 ± 0.36	–

### C-storage and C-sequestration

Significant variation of C-storage was not observed for both the bamboos. However the C-storage corresponds accordingly with the level of aboveground biomass in the different age classes. The ≥ 3 year old age class contributes maximum in the C-storage followed by 2 year old age class and least in the 1 year old age class (Table 4). Total C-storage in BT was 36.34 Mg/ha during 2016 and 64.0 during 2017. In DL it was 50.11 in 2016 and 65.16 in 2017. C-storage in litterfall for both the years was 0.13 Mg/ha and 0.21 Mg/ha in BT and DL, respectively. Rate of C-sequestration was higher in BT with 27.79 Mg/ha/year than DL which have 15.26 Mg/ha/year.

**Table 4:** C-storage in above ground biomass of BT and DL.

	BT( Mg/ha)		DL (Mg/ha)	
	2016	2017	2016	2017
1 year	3.90	7.85	6.12	9.77
2 year	10.7	10.87	6.11	9.17
≥ 3 year	21.74	45.27	37.88	46.22
Total	36.34	64.00	50.11	65.16
C-storage in litterfall	0.08	0.11	0.10	0.11

## Discussion

The culms for both the bamboos have more population in the older age class compared to the younger age class. The distribution pattern was 1:1:3 for 1, 2 and ≥ 3 year old in both BT and DL. The recommendation by Yuming et al.^17^ that maintenance of age class structure of 3:3:3:1 for 1–4 year old bamboo culms for optimum culm production was not observed in the present study. The reason for lower younger culms could be attributed to lack of harvesting of the mature culms as the center was a protected site. Another factor can be attributed to illegal harvesting of the young shoots by the local people for food. Similar pattern of maximum density of older age culms was also observed from Masha bamboo forest in Ethiopia^18^ due to lack of harvesting of the older culms. Nath and Das^19^ reported a stand population structure of 4:3:2:1 in 1–4 year old culms in a bamboo grove in Barak valley of Assam in Northeast India. Since the bamboo stands was in a village, the villagers regularly harvest the old culms, thereby the forest was in an equilibrium state.

Yuming et al.^17^ also suggested that low percentage of young culms, high prevalence of old culms and litterfall biomass similar to or higher than the new shoot produced are indicators of forest degradation. However in the present study the litterfall biomass was very low which indicates that stage to degradation have not reached in the present forest. Therefore for protection from initiation of forest degradation culms ≥ 3 year old should be harvested after proper planning for its utilization.

The average culm density was 14132culms/ha in BT and 17,240 culms/ha in DL showing higher culm density in DL. In the second year study period increase in culm densities were maximum in the ≥ 3 year old age class. The increase in culm density of BT during second year in the ≥ 3 year old age class was 2933culms/ha whereas in DL it was 3840 culms/ha. As the culm density plays an important role in the level of aboveground biomass indicated by the correlation results, the increment in culm density especially in the older age class in the second year can be the determining factor for the amount of aboveground biomass. The present culm densities were higher than 7171culms/ha of *Bambusa vulgaris* from Ghana^20^ (Table 5); from moso bamboo of China which reported a range of 3400–4220 culms/ha^21^; from Majumdar et al.^22^ which reported 1088 culms/ha of *Bambusa tulda* from Tripura Northeast India; 1860 culms/ha of *Bambusa tulda* and 1364 culms/ha of *Dendrocalamus strictus* from Northern India^23^; 7365 culms/ha of village bamboo grove from Assam Northeast India^19^ and 2933 culms/ha of *Bambusa vulgaris* from Bangladesh^24^. However the present range was lower than Singnar et al.^10^ which reported a culm density of 39,075 culms/ha of *Melocanna baccifera* a monopodial bamboo from Assam. The present culm density was comparable with 20,784 culms/ha of *Yushania alpina* from West Amhara, Ethiopia^5^.

**Table 5:** Comparative study of DBH, culm density, aboveground biomass (AGB), C-storage and C-sequestration in different types of bamboo forests.

Sl no.	References	Bamboo species	DBH (cm)	Culm density (culms/ha)	AGB (Mg/ha)	C-storage (Mg/ha)	C-seq (Mg/ha/year)
1	^28^	*Dendrocalamus latiflorus*	–	–	115.4	55.8	11.2
2	^30^	*Phyllostachys pubescens*	8.7	8138	114.6	55.8	10.94
3	^9^	*Phyllostachys makinoi*	–	21,191	105.33	49.81	9.89
4	^7^	Village bamboo grove	6.61	7365		49.11	21.36
5	^6^	*Guadua angustifolia*	16.8	4500	200	100	–
6	^25^	*Schizostachyum pergracile*	–	8600	162.2	78.11	–
7	^24^	*Bambusa vulgaris*	–	2933	97.8	50.44	–
8	^22^	*Bambusa tulda*	–	1088	41.84	20.92	–
9	^23^	*Dendrocalamus strictus*	0.059	1364	12.94	6.47	–
		*Bambusa tulda*	0.086	1860	70.4	35.2	–
		*Bambusa balcooa*	0.091	1860	104.7	52.35	–
10	^30^	*Gigantochloa *sp.	–	7190	–	43.67	–
11	^31^	*Bambusa vulgaris* Schrad	–	2296	66.58	29.70	–
12	^10^	*Melocanna baccifera*	–	39,075	118.4	58.2	–
13	^26^	*Phyllostachys pubescens*	8.63	2589	–	13.96	0.94
14	^20^	*Bambusa vulgaris*		7171	114.97	50.76	8.46
15	^5^	*Yushania alpina*	5.7	20,784	95.4	47.7	–
16	Present study	*Bambusa tulda*	4.95	14,132	100.29	50.17	27.79
		*Dendrocalamus longispathus*	5.70	17,240	132.5	57.63	15.26

The contribution of culm component was maximum with 84–86% followed by branch component with 8.1–8.7% and minimum in leave component with 5–7.1% in both the bamboos. The present range is in the same range with *Schizostachyum pergacile* bamboo form Northeast India^25^ which reported 83.67%, 8.94% and 7.39% contribution by culm, branch and leave components respectively; with mixed bamboo forest from Ghana which had 90.3%, 5.7% and 4.0% contribution of culm, branch and leave components, respectively^20^.

Aboveground biomass was much higher in DL with a total of 115.08 Mg/ha and 150.0 Mg/ha in first year and second year study period respectively whereas, in BT it was 73.58 Mg/ha and 127.03 Mg/ha in first year and second year, respectively. There was a wide gap in aboveground biomass of BT between the 2 years study period especially in the ≥ 3 year old age group with as much as 41.79 Mg/ha. As mentioned above the high increment in culm density of ≥ 3 year old culms in BT during the second year study period was the main reason for such a result. The reason could be higher culm density in the 2 year old age class in BT during the first year study period which ultimately comes under ≥ 3 year old age class in the second year. The culm density of DL was lower in the 2 year old age class in both the study period thereby a wider gap between the 2 years was not observed in aboveground biomass. A difference of only 16.74 Mg/ha in aboveground biomass in the ≥ 3 year old age class between the 2 years was recorded in DL. The positive correlation between aboveground biomass and DBH in BT was also a significant factor for the abrupt increase in aboveground biomass in the second year study period. There was an increase of 0.45, 0.60 and 0.68 cm in the average DBH of BT in the 1, 2 and ≥ 3 year old age class respectively, indicating a maximum increase in the older age class. Whereas in DL, increase in DBH was observed only in 1 year old age class in the second year.

The contribution of the ≥ 3 year old age group to total aboveground biomass was maximum in both the bamboos with 65% and 74% in BT and DL, respectively. Xu et al.^26^ have also reported a similar trend of a maximum contribution of 50% by the 3 year old age group from a moso bamboo forest from Zhejiang province, China. Increase in culm biomass with increase in age was also observed in *Melocanna baccifera* bamboo forest^27^; *Phyllostachys makinoi*^9^ and *Dendrocalamus latiflorus*^28^. The younger culms have more moisture content, as age progresses the level of moisture declines leading to higher contribution in the aboveground biomass.

The present range of aboveground biomass was comparable with Pathak et al*.*^23^ which reported 104.7 Mg/ha from a *Bambusa balcooa* and 70.4 Mg/ha from *Bambusa tulda* forest in Utter Pradesh, Northern India; 162.2 Mg/ha of *Schizostachyum pergacile* from Manipur Northeast India^25^ and 114.6 Mg/ha of moso bamboo from Taiwan^29^. The culm density was much lower in their studies compared to the present study. The present range is also comparable to 105.33 Mg/ha of *Phyllostachys makinoi* from Taiwan^9^ which have almost the same range of culm density (Table 5) with the present work. However it was lower than Quiroga et al.^6^ which reported aboveground biomass of 200 Mg/ha from *Guadua angustifolia* bamboo forest in Bolivia. In their study the DBH of the bamboo was very high with 16.8 cm and a low culm density of 4500 culms/ha.

The C-storage in BT ranged from 36.34 to 64.0 Mg/ha and 50.11 to 65.16 Mg/ha in DL in the 2 years. The results correspond according to the level of aboveground biomass. A comparative study of aboveground biomass, C-storage and rate of C-sequestration is provided in Table 5. The present rate of C-storage in aboveground biomass was higher than 20.92 Mg/ha of *Bambusa tulda*^22^; 6.47 Mg/ha of *Dendrocalamus strictus*^23^ and 13.96 Mg/ha of moso bamboo^26^. In some studies provided in Table 5, the C-storage in aboveground biomass was not given. It was calculated as halve of the aboveground biomass as almost 50% of the biomass is equivalent to C-storage. The rate of C-sequestration was 27.79 Mg/ha/year in BT and 15.26 Mg/ha/year in DL. The present range of C-sequestration was comparable with 18.93–23.55 Mg/ha/year reported by Embaye et al*.*^18^ and 21.36 Mg/ha/year reported by Nath and Das^27^ but higher than most of the studies represented in Table 5. The culm density was very high in the present study, leading to the high rate of C-sequestration in the aboveground biomass. Since the present study area is a protected bamboo reserve harvesting of the older culms was not carried out by the concerned authorities leading to a huge outgrowth of the older culms. The comparative study between the two types of bamboo species indicates that BT has more capability to sequester more amount of C in the aboveground biomass. However it can be suggested that estimation of rate of C-sequestration in aboveground biomass should be carried for a longer duration of years so that large differences in level of C-storage between successive years can be normalized.

From the above findings it can be conclude that density of the culms is an important factor in the study of aboveground biomass of bamboos. In order to maintain a stable ecosystem of bamboo forest harvesting of old culms is important as concentration of older culms would hamper sprouting of new shoots. Moreover the study also showed that aboveground biomass in the stands of *Bambusa tulda* and *Dendrocalamus longispathus* have high potential for sequestration of C. As also suggested by Nath and Das^27^ C-sequestration by bamboo forest can be considered for CDM projects under Kyoto Protocol. It will eliminate poverty and environmental degradation. Therefore initiatives can be taken up by policymakers to utilize the barren lands for plantation of bamboo.
